# COVID-19 and mental health: a multi-country study—the effects of lockdown on the mental health of young adults

**DOI:** 10.1186/s43045-021-00116-6

**Published:** 2021-08-09

**Authors:** Areeba Shaikh, Ellen Peprah, Rawan Hamed Mohamed, Abeeha Asghar, Noor Viresh Andharia, Niel Anthony Lajot, Muhmmad Fazal Hussain Qureshi

**Affiliations:** 1 Youth Center for Research, Karachi, Pakistan; 2 Ziauddin University, 4/B, Shahrah-e-Ghalib, Block 6, Clifton, Karachi, Pakistan; 3 School of Medicine and Dentistry, Kwame Nkrumah University of Science and Technology, Kumasi, Ghana; 4 University of Pécs, Pécs, Hungary; 5 University of Veterinary and Animal Sciences, Sub Campus, Jhang, Pakistan; 6 Osmania University, Hyderabad, India; 7 Silliman University, Dumaguete, Philippines

**Keywords:** Depression, Anxiety, Stress, Coping strategies, COVID-19 pandemic, Young adults

## Abstract

**Background:**

Lockdown conditions due to the COVID-19 pandemic have affected the mental health of people, especially the youth. This study examined the effect of lockdown on mental health of Egypt, Ghana, India, Pakistan, and the Philippines and assessed the coping strategies in practice by youth.

**Results:**

Philippines had the highest mean score in all three domains followed by Egypt, Pakistan, India, and then Ghana. There was a significant association of gender with stress, educational status with depression, and anxiety with stress. Students were associated with anxiety, and the history of close friends/family infected with COVID-19 was found to be associated with depression and stress scores. The most common coping strategy for the Philippines was self-destruction; for Pakistan was religion; and for Egypt, India, and Ghana was acceptance. Using linear regression model, the highest scores observed in all three domains were associated with avoidant coping.

**Conclusions:**

The findings of the study confirm that COVID-19 lockdown has affected the mental health of young adults. In particular, the presence of negative coping strategies used by the youth tends to be an indicator of increased levels of stress, anxiety, and depression and it should be considered when planning interventions within this population.

## Background

According to the South China Morning Post, the first case of the novel coronavirus disease (COVID-19) dates back to 17th November 2019. Since then, over 25.2 million cases have been recorded worldwide, with more than 846,000 deaths [1]. As the world succumbs to the novel coronavirus disease 2019 (COVID-19) pandemic, people may find the new normal stressful and unbearable. The high infectivity of the virus calls for maintaining self-isolation, personal distance, hygiene, and imposing lockdowns to curb the spread. Though proven to be effective, these measures can be overwhelming for individuals and may adversely affect their mental health, such as depression, anxiety, and stress [2–5].

Some researchers have reported that young people are at a higher risk of mental health disorders during the COVID-19 pandemic [6–9]. For adolescents, having social interaction at this stage is an important event; they are energetic and enthusiastic for new day-to-day experiences, making it hard for them to isolate themselves at home during the lockdown [10]. Many researchers have found intense psychological effects in individuals due to the outbreak of pandemics. People may experience feelings as worry about being infected, fear of stigma or isolation, resource instability, self-criticism, and hopelessness are risk factors leading to depression, anxiety, and stress during the lockdown period [11]. Further posit that predictors of mental illness during the pandemic include their marital status, gender, parenting status, employment status, comorbidities, religion, and family size. The authors, however, declared that the causality cannot be firmly established [12].

Additionally, coping could be considered a conscious, unconscious, intentional, or programmed response to stress by an individual [13–15]. Eisenberg et al. broadly classify coping strategies into two: avoidant coping and approach coping, where approach coping is more likely to lead to a favorable outcome than avoidant coping. Other classifications of coping strategies include problem-focused, emotion-focused, cognitive, and behavioral, and neutral coping mechanisms [16].

Literature reports conflicting results for the most common coping strategies used during the COVID-19 pandemic [17]; problem-focused coping, seeking social support, and making a positive appraisal of the situation as the most common coping strategies used by their respondents [17]. Others named are physical activity/exercise, online self-guided counseling sessions [18], gaming [19], and exergaming [20]. Umucu et al. found positive associations between respondents’ perceived stress and coping mechanisms like behavioral disengagement, self-distraction, substance use, venting, planning, religion, and self-blame. They further found associations between participants’ wellbeing and other coping mechanisms like humor, religion, active coping, emotional support, and denial [21].

This study is highly relevant to the Egyptian, Ghanaian, Indian, Pakistani, and Filipino contexts due to the lack of information on depression, anxiety, and stress in the general youthful population. Though the existing literature on mental health in these countries holds relevance for other demographics [2, 22–25]. However, there is a general shortage of information on mental health among young people within the context of COVID-19. This study assesses the mental health of young adults in Egypt, Ghana, India, Pakistan, and the Philippines during the COVID-19 lockdown. It further reviews the coping mechanisms used by the youth of these countries.

## Methods

### Participants and procedure

The sample size was calculated using OpenEpi and was found to be 364 at a 95% confidence interval with a 5% margin of error. A total of 526 young adults were conveniently sampled in a cross-sectional study by responding to a web-based survey. A link to the semi-structured survey was posted on different social media platforms. The questionnaire was in the English Language. Data was collected for a period of 8 weeks from June 2020 to July 2020. The respondents were asked to recall their experiences from the first month of the lockdown and determine each statement related to them. The research was conducted by the Helsinki Declaration as revised in 1989 and was approved by the ethical review board of Youth Center for Research, Karachi, Pakistan. (Reference Number: 2020SRPPH).

### Inclusion criteria

- The participants must range between the age group of 18 to 30 years.
- Participants must have completed the voluntary consent form.
- Participants must be able to read and understand English.
- Participants must be located within the geographical boundaries of Pakistan, India, Ghana, Egypt, and the Philippines.

### Exclusion criteria

- The participants who have any severe medical illness or severe mental condition and mental retardation will be excluded.
- Participants who are above the age of 30.

### Measuring/study tool

#### Socio-demographic characteristics

Age, country, gender, marital status, households they live in, educational status, whether they were current students, the academic year they were currently in, religion, employment status, any chronic medical condition, having pets, themselves or any close one being infected by COVID-19, death of their known ones due to COVID-19, and no. of children they had, if any.

#### Depression, Anxiety, and Stress Scale (Lovibond and Lovibond 1995 [26])

The DASS-21 is the simplified DASS version created by Lovibond and Lovibond to assess individual symptoms of depression, anxiety, and stress. The questionnaire consists of 21 items with 4 answer options: 0 “did not apply to me at all—never,” 1 “applied to me to some degree, or sometimes—sometimes,” 2 “applied to me to a considerable degree, or to a good part of the time—so often” to 3 “applied to me very much, or most of the time—almost always.”

The respondents were asked to recall experiences over the past 7 days and determined how each statement shows relevance. Scores can be measured on three subscales identified as DASS-21-Depression (DASS-21-D), DASS-21-Anxiety (DASS-21-A), and Stress (DASS-21-S). In each of the subscales, there are seven items; and the score of each subscale ranges between 0 and 21. DASS-21’s reliability demonstrates that it has extremely good Cronbach’s alpha values of 0.81, 0.89, and 0.78, respectively, for the depressive, anxiety, and stress subscales. It also shows good internal consistency, discriminative, concurrent, and convergent validities. Thus, DASS-21 is reliable, easy, and valid to administer, and it has commendable psychometric properties [26].

#### The Brief-COPE (Carver et al. 1997)

The Brief-COPE is a 28-item self-report questionnaire developed from the extended version of the original 60-item COPE scale [27] (Carver et al. 1997) which is designed to assess a wide range of coping responses for a wide range of adversities including all diseases, stress, natural disasters, assaults, etc. among adults. The ems on the scale are rated on the four-point Likert scale, ranging from “I have not been doing this at all” (score 1) to “I have been doing this a lot” (score 4). The higher score in this study reflects greater coping strategies employed by the respondents. In total, the scale covers 14 dimensions. The scale evaluates an individual’s primary styles of coping as either approach coping or avoiding coping. Additionally, the following subscales are reported: self-distraction, active coping, denial, drug use, use of emotional support, use of instrumental support, behavioral disengagement, venting, constructive reframing, preparation, humor, acceptance, faith, and self-blame. Every dimension has two items [28].

### Data analysis

Data was entered into IBM SPSS version 20.0 (Armonk, NY, USA). Once this prerequisite was met, the linear regression model was performed. Chi-square test was applied on the skewed categorical data while remaining analysis was done via ANOVA and Tukey’s honest significant difference test. The tests were two-tailed, and significance level chosen was a *p* value < 0.05. Descriptive frequencies were also applied to assess some sociodemographic variables and highlight notable trends.

## Results

**Fig. 1:**
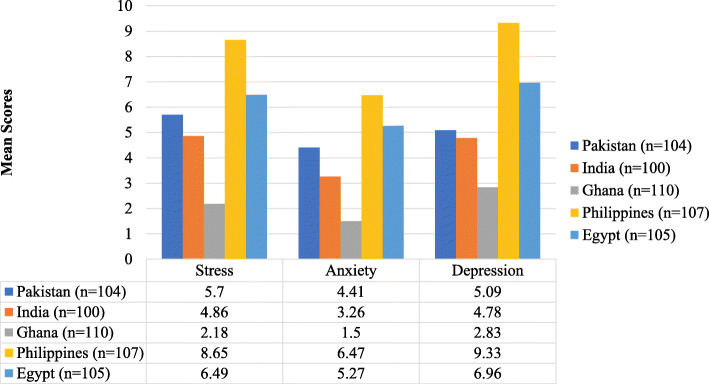
Mean stress, anxiety, and depression score among countries

The mean age of respondents was 22.3 years with a standard deviation of 3.1 years. Out of 526 respondents, 60.3% (*n* = 317) were females and 39.7% (*n* = 206) were males. Sample comprises of young adults of Ghana (20.9%, *n* = 110), Philippines (20.3%, *n* = 107), Egypt (19.7%, *n* = 105), Pakistan (19.7%, *n* = 104), and India (19.1%, *n* = 100). The depression, anxiety, and stress scores were calculated for each country. The Philippines had the highest mean score in all three domains, and Ghana had the lowest as shown in Fig. 1. Further, prevalence of different levels of stress, anxiety, and depression is shown in Table 1.

**Table 1:** Prevalence of stress, anxiety, and depression levels among Egypt, Ghana, India, Pakistan, and Philippines

Country	Condition	Normal		Mild		Moderate		Severe		Extremely severe	
		n	%	n	%	N	%	n	%	n	%
Egypt (*n* = 105)	Stress	66	62.9	13	12.4	15	14.3	08	7.6	03	2.9
	Anxiety	38	36.2	25	23.8	16	15.2	11	10.5	15	14.3
	Depression	34	32.4	26	24.8	17	16.2	15	14.3	13	12.4
Ghana (*n* = 110)	Stress	99	90.1	04	3.6	04	3.6	01	0.9	02	1.8
	Anxiety	97	88.2	05	4.5	03	2.7	03	2.7	02	1.8
	Depression	84	76.4	11	10	08	7.3	05	4.5	02	1.8
India (*n* = 100)	Stress	77	77	06	6	09	9	05	5	03	3
	Anxiety	59	59	19	19	09	9	06	6	07	7
	Depression	58	58	16	16	13	13	04	4	09	9
Pakistan (*n* = 104)	Stress	71	68.3	10	9.6	08	7.7	10	9.6	05	4.8
	Anxiety	57	54.8	11	10.6	13	12.5	12	11.5	11	10.6
	Depression	54	51.9	12	11.5	26	25	05	4.2	07	6.7
Philippines (*n* = 107)	Stress	51	47.7	13	12.1	15	14	12	11.2	16	15
	Anxiety	38	35.5	16	15	16	15	13	12.1	24	22.4
	Depression	29	27.1	15	14	21	19.6	14	13.1	28	26.2

A chi-square test was further applied to determine the association between depression, anxiety and stress scores, and demographic variables. There was no association found between depression and religion (*p* value = 0.540), year of study (*p* value = 0.403), household system (*p* value = 0.466), having pets (*p* value = 0.164), relationship status (*p* value = 0.66), or employment (*p* value = 0.366). There was no significant association found between anxiety and religion (*p* value = 0.579), year of study (*p* value = 0.093), household system (*p* value = 0.753), having pets (*p* value = 0.985), relationship status (*p* value = 0.298), or employment status (*p* value = 0.400). There was also no association found between stress and religion (*p* value = 0.339), year of study (*p* value = 0.948), household system (*p* value = 0.758), having pets (*p* value = 0.517), relationship status (*p* value = 0.69), or employment status (*p* value = 0.566).

On the other hand, there was a significant association between gender and stress, educational status and depression, anxiety and stress, students and anxiety, and history of close friend/family infected with COVID-19 and depression and stress scores as shown in Table 2.

**Table 2:** Association of stress, anxiety, and depression scores with demographics

Variable	Stress						Anxiety						Depression					
	Normal	Mild	Moderate	Severe	Extremely severe	*p* value	Normal	Mild	Moderate	Severe	Extremely severe	*p* value	Normal	Mild	Moderate	Severe	Extremely severe	*p* value
Gender																		
Male	154	16	21	11	7	0.031	123	28	23	16	17	0.178	108	34	37	12	17	0.179
Female	211	30	30	25	21		166	48	33	29	42		151	46	41	31	42	
Educational status																		
High School	15	3	3	5	5	0.008	08	06	02	04	11	0.000	08	06	06	03	08	0.013
Undergraduate	193	34	30	17	11		150	50	35	27	23		130	54	49	23	29	
Graduate	122	7	12	13	9		100	14	16	11	22		96	12	23	15	17	
Post-Graduate	35	7	6	1	3		31	06	04	03	03		25	08	07	02	05	
Are you currently a student?																		
Yes	240	35	37	23	16	0.401	181	60	41	33	36	0.045	167	61	61	26	36	0.168
No	125	11	14	13	12		108	16	16	12	23		92	19	24	17	23	
Close friend/family member infected with COVID-19																		
Yes	133	24	25	19	13	0.047	98	35	27	22	31	0.016	87	35	37	25	29	0.10
No	233	22	26	17	15		191	41	30	23	28		172	45	48	18	30	

Data was divided according to different nationalities and sociodemographic variables were analyzed to determine their association with stress, anxiety, and depression scores. Participants from the Philippines have significant association of high depression scores and female gender (*p* = 0.035), participants with relationship status of bachelor have significant association with high anxiety scores (*p* = 0.026), participants living in nuclear family has significant association with high depression scores (*p* = 0.047), and lastly participants in their final year of study has significant association with higher anxiety scores (0.045).

Participants from Pakistan have significant association of high depression scores and relationship status of bachelor (*p* = 0.006) and participants having friend and family infected with COVID-19 has significant association with high anxiety scores (*p* = 0.037).

Participants from India living in nuclear family has significant association with high anxiety scores (*p* = 0.025), participants having death within friends and family due to COVID-19 has significant association with high depression scores (*p* = 0.036) and stress scores (*p* = 0.041) and lastly students have significant association with higher anxiety scores (0.006).

Participants from Ghana living in nuclear family has significant association with high anxiety scores (*p* = 0.012) stress scores (*p* = 0.000) and depression scores (*p* = 0.000), participants having death within friends and family due to COVID-19 has significant association with high depression scores (*p* = 0.033) and anxiety scores (*p* = 0.000).

Participants from Egypt, who are currently students, have significant association with high depression scores (*p* = 0.026), and lastly youth in their 1st year of study has significant association with higher anxiety scores (0.000) and depression scores (*p* = 0.029).

**Fig. 2:**
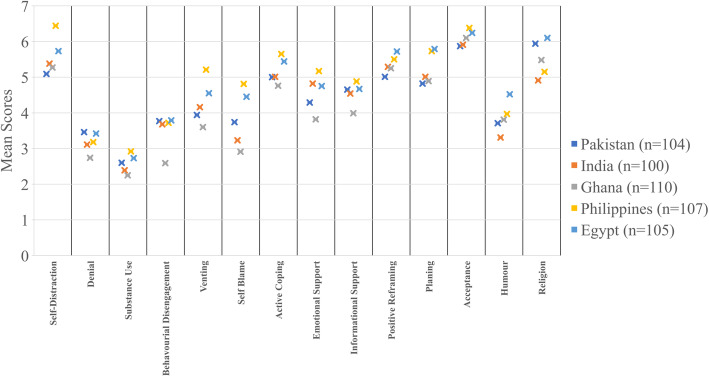
Mean coping strategies score among countries

Figure 2 shows the different coping strategies and their distribution country-wise based on their mean scores (2 being minimum while 8 being maximum). The most common coping strategy for the Philippines was self-destruction, for Pakistan, religion and for Egypt, India, and Ghana, acceptance.

A linear regression model comparing stress, anxiety, and depression and their associations with coping strategies was applied. Coping strategies were divided into three broader categories: avoidant coping, approach coping, and neutral coping. The highest scores observed in all three domains were associated with avoidant coping as shown in Table 3.

**Table 3:** Linear regression model comparing stress, anxiety, depression scores with coping mechanisms

Coping mechanism	Stress scores		Anxiety score		Depression score	
	B	*p* value	B	*p* value	B	*p* value
Avoidant coping						
Self-destruction	0.334	0.011	0.186	0.101	0.377	0.005
Denial	− 0.219	0.110	0.012	0.922	− 0.110	0.433
Substance abuse	0.137	0.331	0.523	0.000	0.258	0.073
Behavioral Disengagement	0.759	0.000	0.540	0.000	0.748	0.000
Venting	0.577	0.000	0.312	0.012	0.433	0.003
Self-blame	0.945	0.000	0.757	0.000	1.207	0.000
Approach coping						
Active coping	− 0.061	0.670	0.184	0.140	− 0.205	0.162
Emotional support	− 0.031	0.820	− 0.053	0.653	− 0.122	0.381
Informational support	0.163	0.240	0.095	0.426	0.103	0.467
Positive reframing	− 0.268	0.049	− 0.141	0.230	− 0.307	0.027
Planning	0.138	0.350	0.024	0.851	0.043	0.772
Acceptance	0.041	0.762	− 0.112	0.338	0.212	0.124
Neutral coping						
Humor	0.054	0.598	0.064	0.469	0.040	0.701
Religion	− 0.195	0.051	− 0.107	0.216	− 0.299	0.003

## Discussion

This multi-country cross-sectional study has evaluated Egypt, India, Ghana, Pakistan, and the Philippines. However, these 3rd world countries have suffered the most during the period of the pandemic. Considering Pakistan, India, and Ghana are multi-ethnic countries, the compliance and response to the lockdown and the capability of populations to protect their health have varied right through. Muslim majority countries have encountered a considerable burden of COVID-19 infection [29]. According to Islamic teachings, family kinship, looking after the elderly, praying and eating meals in a congregation, and sharing the same utensils are commonly practiced, which may have increased the spread of COVID-19 among Muslim communities. Additionally, the Philippines is a low economic country with inadequate health information and hospital facilities hence leaving its people prone to the current pandemic crisis.

The present study assessed the composite scores of depression, anxiety, and stress among the young adult population of Egypt, India, Ghana, Pakistan, and the Philippines, after the onset of lockdown during the COVID-19 pandemic. We also assessed the association of coping strategies mainly deployed during the lockdown with depression, stress, and anxiety. It has been hypothesized that COVID-19 extended lockdown increases depressive symptoms, stress, and anxiety in young adulthood.

Females are more likely to encounter mental health issues during the coronavirus pandemic. Positive coping strategies would help decrease depression, stress, and anxiety levels among early adults. Developing countries are in the lead of facing mental health challenges out of the lockdown policies.

The analysis indicated increased percentages of depression among the Philippines 8.7%, Egypt 6.6%, Pakistan 4.8%, India 4.7%, and Ghana 2.5%. Following these findings, Lee et al. found that Filipino students expressing depressive symptoms and had a statistically significant association with smoking, frequency of drinking, not living with biological parents, dissatisfaction with one’s financial condition, and level of closeness with parents and peers [30]. Similarly, using the DASS-21 scale on a sample of 2178 Egyptian participants, Fawzy and Hamed recorded 65% of young adults suffering from depression [31]. Another study in Pakistan analyzed normal (65.9%), mild (9.10%), moderate (9.12%), and severe (15.90%) depression prevalence during the COVID-19 lockdown [32]. Sandesh et al. also reported that 81 out of 112 Pakistani respondents suffered from moderate to extremely severe depression with a mean score of 18.12 ± 10 [33]. The levels of depression were also prevalent in a study conducted on the Indian population, as 25% of the participants expressed depressive symptoms [34]. The studies mentioned above have shown a significant association of depression with binge drinking and smoking [30, 34]. The negative emotional challenges experienced during the lockdown may have caused individuals to adapt to escaping mechanisms to cope with the new normal.

The present study highlighted the percentages of stress, 8.0%, 6.2%, 5.5%, 4.9%, and 2.0%, in the Philippines, Egypt, Pakistan, India, and Ghana. This supports the findings of Fawzy and Hamed, in which Egyptian adults recorded high scores on the Depression Anxiety Stress Scale (DASS 21) with remarkably high scores on the stress subscale (DASS-21-S) during the pandemic [31]. It equally conforms to what El Zoghby, Soltan, and Salama indicated that out of many psychological problems, Egyptian adults encountered severe stress with different types [11]. These psychological challenges can be attributed to the lack of practicing active coping during COVID-19. In contrast to our findings, two studies showed elevated stress levels during the pandemic [34, 35]. Furthermore, the anxiety levels were considerably less than the levels of depression and stress evaluated from this study. The reported percentages in the Philippines, Egypt, Pakistan, India, and Ghana were 6.0%, 5.0%, 4.2%, 3.3%, and 1.4%, respectively. Another study reported analogous findings, where the majority of students, aged 22–26, suffered from anxiety and had adapted to common personal coping strategies such as individual protective measures throughout lockdown [36]. Ahmad et al. conducted a study on the Indian population aged 15 years and above and reported the prevalence of anxiety in 25.4% of 398 respondents [12]. One reason could be that different countries have faced unprecedented events throughout the pandemic, which may have increased psychological distress severity [37]. While in this study, the young Ghanaian adults recorded low levels of overall psychological distress. These lower scores may be attributed to the respondents being accustomed to adapt cognitive coping strategies [38].

The study also demonstrated that with the greater adaptation of avoidant coping mechanisms during COVID-19 lockdown, the severity of depression, anxiety and, stress has worsened. Those populations with higher levels of depression, anxiety, and stress have adapted avoidant coping mechanisms; self-distraction, denial, substance abuse, behavioral disengagement, venting, and self-blame. In comparison, those with lower levels have adapted approach coping mechanisms; acceptance, active coping, emotional and informational support, positive reframing, and planning or neutral coping such as religion and humor. However, emotion-focused coping mechanisms such as crying, being angry, yelling, drinking, or smoking harm psychological health [39]. In contrast to our findings, wherein religious coping was categorized as a neutral coping strategy, a previous study stated that frequently praying may result in an elevated risk of mental health issues, but to a lesser extent [39].

Self-distraction is identified in the current study as a common type of avoidant coping mechanism—demonstrating a positive relationship with psychological distress and a negative relationship with psychological wellbeing. Linear regression showed a significant association of avoidant coping with stress, anxiety, and depression during the lockdown. Notably, the complete abstention of avoidance coping can result in psychological inflexibility—that is, unable to respond effectively to situational demands-which is necessary to facilitate a valued outcome [28]. By deploying effective coping strategies during the pandemic, the individuals try to alleviate stress, anxiety, and depression.

In addition, the levels of stress and depression were significantly associated with the concerns of respondents who have had people infected in their surroundings. Results collected from a study conducted in America identified mean depressive symptoms with mild symptoms for anxiety among the respondents, while stress levels did not yield any significance [40].

On studying the association between sociodemographic characteristics and mental health issues, stress, anxiety, and depression were not associated with religion, employment status, or relationship status. The results were also consistent with the findings of a study that showed no effect of relationship status on the mental health of young adults [41].

Nonetheless, the female gender was associated with stress, and educational status was associated with stress, anxiety, and depression [11, 31, 42]. On the other hand, Arnout et al. also reported that neither academic class nor gender was associated with psychological stress, which contradicts the findings of this study [41].

Another research in India found an association between female gender, employment status, religion, relationship status, and anxiety [12]. Contrary to what was indicated by the results of our study. In accordance with our findings, a study reported no effect of employment status on anxiety among Filipino young adults. However, they found no effect of the educational level on anxiety, contrary to our study results [43].

Our study revealed that young adults between the ages of 20 and 25 recorded the highest scores of stress, depression, and anxiety in terms of age. Consistently, Fawzy and Hamed found that those between the ages of 18 and 30 expressed a higher psychological impact than older participants [31]. In addition to what was mentioned by Varshney, Parel, Raizada, and Sarin that younger adults are more prone to mental health issues during the coronavirus pandemic [42]. Nevertheless, our results contradict the finding evaluated in this research study that age is a positive predictor of anxiety and depression in Ghana during COVID-19. Older adults are vulnerable to higher psychological distress [44].

Studentship was also associated with anxiety. In line with this, an Egyptian study emphasized the association between studentship and mental health issues, particularly anxiety, in medical students [9].

This study adds to the current research on the COVID-19, particularly in Africa and Asia. It covered the general population, not just the medical one, as being widely investigated in prior studies. Additionally, it sheds light on the essence of researching coping strategies during the coronavirus pandemic and the associated cultural differences while addressing mental health. Given the contradictory findings between the literature and the current research on COVID-19, future research is recommended to

1. Include a larger sample size to maintain a more accurate representation of the target population.
2. Maintain a balanced sample in terms of gender and a broader demography investigation.
3. Use more detailed questionnaires for more reliable data.
4. Consider conducting qualitative research.

Drawing on the findings of this research, we also recommend the decision-makers to

1. Use telehealth service in Egypt, Ghana, India, Pakistan, and particularly the Philippines to facilitate mental health services and increase the prospects of delivering the needed psychological support to young adults during the coronavirus pandemic.
2. Boost the interventions and mental health awareness campaigns to stress the necessity of mental health care related to physical health, daily production, academic performance, and other life domains.
3. Initiate national COVID-19 helpline to encourage people to seek the help they need.

Looking into the limitations of our study, they include relatively small sample size, using an online survey that requires participants to be literate and healthy, which poses an issue of reaching the illiterate or unhealthy population. Hence, this limits our capacity to generalize the results for the uneducated or people with psychiatric illness. Another reason for the small sample size is that this paper resulted from an 8-week summer research program. Equally important, controlling the participants’ characteristics through an Internet-based questionnaire was challenging, provided that some participants could deviate from the ethical guidelines and misrepresent themselves. Moreover, this research was limited to five nationalities as the authors belong to these five countries and can represent their cultural and traditional aspects more comprehensively. Finally, the online survey response rate was relatively low compared to other methods that might have yielded an 11% higher response rate [45].

## Conclusion

In this sample of relatively healthy youth, the highest scores on stress, anxiety, and depression were found in the Philippines, followed by Egypt, Pakistan, and India. The lowest scores were found in the Ghanaian population. The study also revealed significant gender differences. Female participants displayed higher levels of stress than male participants. Undergraduates showed significantly higher levels of stress, anxiety, and depression. Participants who were still studying scored high on anxiety, and those that had a history of friends/family members being infected with COVID-19 showed higher levels of stress and depression. The research findings also indicated different coping strategies used by respondents in the countries mentioned above. A positive correlation was found between avoidance coping and levels of stress, anxiety, and depression. There was a significant association between negative coping mechanisms and higher levels of stress, anxiety, and depression.

In contrast, positive coping strategies indicated lower levels of stress, anxiety, and depression. Ultimately, the findings of the study confirm that COVID-19 lockdown has affected the mental health of young adults. In particular, the youth’s presence of negative coping strategies tends to indicate increased levels of stress, anxiety, and depression, and it should be considered when planning interventions within this population.

## Data Availability

The datasets used and/or analyzed during the current study available from the corresponding author on reasonable request.

## Abbreviations

COVID-19: Coronavirus disease 2019
DASS: Depression, Anxiety and Stress Scale
SPSS: Statistical Package for the Social Sciences
ANOVA: Analysis of variance
